# Reduction of Splenic Immunosuppressive Cells and Enhancement of Anti-Tumor Immunity by Synergy of Fish Oil and Selenium Yeast

**DOI:** 10.1371/journal.pone.0052912

**Published:** 2013-01-22

**Authors:** Hang Wang, Yi-Lin Chan, Tsung-Lin Li, Brent A. Bauer, Simon Hsia, Cheng-Hsu Wang, Jen-Seng Huang, Hung-Ming Wang, Kun-Yun Yeh, Tse-Hung Huang, Gwo-Jang Wu, Chang-Jer Wu

**Affiliations:** 1 Department of Food Science and Center of Excellence for Marine Bioenvironment and Biotechnology, National Taiwan Ocean University, Keelung, Taiwan; 2 Graduate Institute of Medical Sciences, National Defense Medical Center, Taipei, Taiwan; 3 Genomics Research Center, Academia Sinica, Taipei, Taiwan; 4 Division of General Internal Medicine, Mayo Clinic, Rochester, Minnesota, United States of America; 5 Institute of Biomedical Nutrition, Hung Kuang University, Taichung, Taiwan; 6 Division of Hemato-Oncology, Department of Internal Medicine, Chang Gung Memorial Hospital, Keelung, and College of Medicine, Chang Gung University, Kaohsiung, Taiwan; 7 Division of Hemato-Oncology, Department of Internal Medicine, Chang Gung Memorial Hospital, Kweishan, and College of Medicine, Chang Gung University, Kaohsiung, Taiwan; 8 Department of Traditional Chinese Medicine, Chang Gung Memorial Hospital, Kweishan, and Graduate Institute of Clinical Medical Sciences, College of Medicine, Chang Gung University, Kaohsiung, Taiwan; Mie University Graduate School of Medicine, Japan

## Abstract

Growing evidence has shown that regulatory T cells (Tregs) and myeloid-derived suppressor cells (MDSCs) abnormally increase in cancer cachectic patients. Suppressions of Tregs and MDSCs may enhance anti-tumor immunity for cancer patients. Fish oil and selenium have been known to have many biological activities such as anti-inflammation and anti-oxidation. Whether fish oil and/or selenium have an additional effect on population of immunosuppressive cells in tumor-bearing hosts remained elusive and controversial. To gain insights into their roles on anti-tumor immunity, we studied the fish oil- and/or selenium-mediated tumor suppression and immunity on lung carcinoma, whereof cachexia develops. Advancement of cachexia in a murine lung cancer model manifested with such indicative symptoms as weight loss, chronic inflammation and disturbed immune functionality. The elevation of Tregs and MDSCs in spleens of tumor-bearing mice was positively correlated with tumor burdens. Consumption of either fish oil or selenium had little or no effect on the levels of Tregs and MDSCs. However, consumption of both fish oil and selenium together presented a synergistic effect-The population of Tregs and MDSCs decreased as opposed to increase of anti-tumor immunity when both fish oil and selenium were supplemented simultaneously, whereby losses of body weight and muscle/fat mass were alleviated significantly.

## Introduction

Cancer cachexia often develops in cancer patients and is the primary cause to high morbidity and mortality in advanced cancers [1]. Though the definition of cachexia remains unclear, common features for this chronic abnormal catabolism include progressive and involuntary weight loss, wasting, asthenia, fatigue, etc [1]. Causes such as tumor-derived factors, therapeutic strategy, nutritional status, age, stress and depression all are linked to progression of cachexia, which manifests as chronic inflammation and impaired immune responsiveness [2]. Immune suppression promotes disease progression and complications, thereby resulting in suboptimal treatment (e.g. surgery, chemotherapy or radiotherapy), and poor quality of life and prognosis [3].

The myeloid-derived suppressor cells (MDSCs) and regulatory T cells (Tregs) of immune suppressive cells recently attract considerable attention as they are implicated in tumor immune escape [4], [5]. MDSCs represent a heterogeneous population of myeloid cells, consisting of immature macrophages, granulocytes, dendritic cells and other myeloid cells at earlier stages of differentiation with common high expressions of CD11b and Gr-1. MDSCs may suppress adaptive and innate immunity through influencing T cell activation [5], NK cell cytotoxicity [6], and Tregs induction [7]. As to Tregs, they represents a small fraction of cells in the overall CD4^+^CD25^+^ T cell population, mediating immune suppression via a cell-cell contact mechanism with a characteristic high level of FOXP3 [8]. Accumulation of MDSCs and Tregs is considered to promote the tumor immune escape, underlining both types of cells an ideal treatment target in immunotherapy.

Prevention and treatment of cancer cachexia have been a standard regimen in cancer therapy. Adequate supplementation of nutrients is beneficial to cancer patients. For example, nutritional supplements may help stop or reverse nutritional decline and offset dysfunction of immune system, thereby ensuring a better clinical outcome towards a good quality of life. Fish oil (fo) derived from omega-3 polyunsaturated fatty acids (PUFAs), particularly eicosapentaenoic acid (EPA), has been known able to prevent nutritional deterioration in cancer patients through inhibiting tumor growth, cancer cachexia progression, and inflammation [9]. Inasmuch as EPA supplementation (EPA total parenteral nutrition (TPN)) improves immunologic parameters for those patients who received postoperative chemo-radiation therapy against esophageal cancer [10] or who underwent thoracotomy for esophageal cancer, experienced less stress-induced immunosuppression [11], such beneficial effects of EPA prompted us to utilize PUFAs in perioperative immunonutrition for a better prognosis. Inflammation and immune suppression in cancer patients with cachexia may be modulatable through supplementing anti-inflammatory and anti-tumor immune nutrients [12].

Selenium (Se) physiologically acts as a potent nutritional anti-oxidant in a fashion of selenoproteins. Roth et al. [13] suggested that Se (50 µg) would strengthen positive immune responses and thereby lower down cancer incidences. Kiremidjian-Schumacher and Roy [14] reported that lymphocyte activities may be fortified with supplementation of selenium, whereby cytotoxic T lymphocyte (CTL)-driven tumorolysis, mitogen-induced proliferation of lymphocytes, and mixed lymphocyte reaction (MLR) proliferation will be enhanced markedly. Significant reduction of cell-mediated immunity in the pre-cachectic state was revealed recently (suggesting an impaired immunity prior to weight loss) [15], while the mechanism exerted through Se-supplementation remains elusive. What was known is supplementation with fish oil (EPA) may reduce production of proinflammatory cytokines, ameliorating cancer-induced weight loss [16]. On this basis, we proposed both fish oil and selenium may have a synergistic effect to neutralize the cancer-induced immune suppression.

In this report, we studied the mechanism of the lung carcinoma-induced immune suppression and the effects of individual and/or combined nutrients on the tumor-induced immune dysfunction. In order to tackle the stated issue, we first developed a stable mouse lung cancer model–immunocompetent BALB/c mouse to validate that cachectic symptoms are associated with immune responses in lung cancer. We further demonstrated that dietary supplementation with fish oil and selenium can markedly reduce the number of immunosuppressive cells in spleens in the tumor-bearing mice but affecting no or little on other immunocytes.

## Materials and Methods

### Cells and cell culture

Line-1 cells from a BALB/cByJ alveolar lung carcinoma (provided from Dr. John Yuhas) were adapted to tissue culture [17]. Line-1 and YAC-1 lymphoma cell line (ATCC-TIB-160) were cultured in RPMI Medium 1640 (Gibco, New York, USA) supplemented with 10% fetal bovine serum (Gibco, New York, USA), and cultured at 37°C in a humidified incubator supplied to 5% of the atmosphere with CO_2_.

### Mice and tumor model

Male BALB/cByJ mice (6–7 weeks) were obtained from the National Laboratory Animal Center (Taipei, Taiwan). Mice were individually housed in a climate controlled room (12∶12 dark-light cycle with a constant room temperature of 21±1°C). Mice underwent at least 4-day adjustment to new environment and diet before treatments were performed. Mice were allowed free access to water and food (laboratory rodent diet, labdiet 5001, USA). After acclimatization, mice were divided into weight-matched groups. In the first experiment (experiment A), mice were inoculated subcutaneously (s.c.) with the suspension of line-1 tumor cells (1×10^5^) on day 0. A control group was injected with 0.1 ml of sterile saline solution. The second experiment (experiment B) was designed to test the effect of single or combined nutrients added to the background diet (addition of fish oil (fo) and/or selenium yeast (se)). Supplementation was administered orally with experimental diet (1 g/mice/day) after inoculation of 5×10^4^ tumor cells, while the control mice received background diet (1 g/mice/day). After inoculation of tumor cells or PBS, body mass, food intake and tumor size were measured four times a week. Tumor growth was assessed using a caliper: recording the inner diameter between the two rulers for each tumor. The tumor volume was calculated using the following equation: tumor volume (mm^3^) = width×length^2^/2. Animals were sacrificed by the CO_2_ inhalation method on day 35 (experiments A) and 42 (experiments B). Organs were removed and weighed, and stored at −20°C for further analysis. The experimental procedures were approved by the Animal Ethical Committee and followed the principles of good laboratory animal care. The animal experiments also complied with the guidelines for the maintenance and handling of experimental animals established by the Institutional Animal Care and Use Committee (IACUC) of the National Taiwan Ocean University of College of Life Sciences (permission number: 99014). The carcass weight was calculated by subtracting the tumor weight from the body weight. Final body weight gain was calculated from the difference between the carcass weight and its initial weight.

### Experimental diets (category B experiment)

The mice in the category B experiment were daily supplemented with 63 mg fats (100% soy oil), 514 mg carbohydrates and 194 mg proteins as a background diet (Ethan Nutraceutical Company Ltd., USA). The experimental diets included additional 20 mg fish oil (contained 11 mg EPA and DHA) and/or 0.69 µg selenium yeast. Fish oil and selenium yeast were obtained from Ethan Nutraceutical Company Ltd. (USA). Mice were divided into five weight-matched groups: 1) Control receiving background diet (Con); 2) tumor-bearing mice receiving background diet (TB-Con), 3) tumor-bearing mice receiving background diet plus fish oil (TB-fo), 4) tumor-bearing mice receiving background diet plus selenium yeast (TB-se), and 5) tumor-bearing mice receiving background diet plus both fish oil and selenium yeast (TB+fo+se).

### Levels of serum albumin and cytokines

The levels of serum albumin were measured for experimental mice using the SPOTCHEM EZ SP-4430 dry chemical system (Arkray, Kyoto). Cytokines in serum were measured using the OptEIA™ ELISA Set (BD, Canada, USA) for mouse TNF-α, IL-6 and IL-1β. Optical density was recorded using a μQuant spectrophotometer (Bio-Tek Instruments, Winooski, USA).

### Splenocyte isolation and flow-cytometric analysis

Splenocytes were isolated by centrifugation (300 g); red blood cells were lysed using the Gey's reagent (0.829 g NH4Cl, 0.1 g HCO_3_ and 3.72 mg Na_2_EDTA in 100 ml ddH_2_O). For determining phenotypes of splenocytes isolated from spleens, cells were stained with an appropriate combination of anti-CD3ε (100306; Biolegend), anti-CD4 (100516; Biolegend), anti-CD8a (100714; Biolegend), anti-CD19 (115508; Biolegend), anti-Gr-1 (108416; Biolegend), anti-Ly6G (127613; Biolegend) or anti-CD11b (101212; Biolegend) after blocking of the Fc receptor with anti-CD32/CD16 (BD Biosciences) at 4°C. For T regulatory cells staining, cells were incubated with anti-CD4 and anti-CD25 (102006; Biolegend) for 30 minutes, followed by fixing in 2% paraformaldehyde, permeabilizing with Perm/Wash buffer (BD Biosciences), and staining with anti-Fox-p3 (320008; Biolegend). For determining NK cytotoxicity, cells were isolated from mouse spleens (regard as effector cells). The target cells (line-1 and YAC-1) were stained with DIOC18 (10 µl per 1×10^6^ cells) for 20 min at 37°C. The cells then were washed twice with a buffer solution and then resuspended in a complete culture media at a concentration of 1×10^6^ cells/ml. The target and effector cells (splenocytes) were prepared by making co-cultured cells in ratios of E∶T = 5∶1, 20∶1, and 40∶1. These co-cultures were incubated for 4 hours, and centrifuged at 250 g for 5 min through alternate washes and suspensions; supernatants were discarded. The cells then were labeled with propidium iodide (PI, 2 µl/per test) and incubated at room temperature in dark. Analysis was performed using FACScan (BD Biosciences). For characterization of cell types, a large gate was set to include monocytes and lymphocytes for forward scatter vs. side scatter.

### RNA extraction and RT-qPCR

Total tumor RNA was extracted with a commercially available RNA mini kit (Qiagen); cDNA was synthesized using the M-MLV reverse transcriptase (Promega) and the oligo-dT15-primer (Promega). Real-time qPCR primers were designed using the Primer3 webware; electrophoresis was performed to verify DNA products. Reactions were run on the Bio-Rad iCycler iQ system in the presence of the Sybr-Green PCR mix (iCycler iQ Real Time PCR Detection System, Bio-Rad). The comparative threshold cycle (C_T_) method was used to calculate the relative expressions [18]. For quantification of gene expressions, the values of target genes were normalized by the value of the endogenous reference GAPDH. The quantity of the target gene relative to a calibrator (normal pool expression) is given by: 2^−ΔΔC^_T_ [ΔC_T_ = C_T_(target gene)−C_T_(GAPDH); ΔΔC_T_ = C_T_ for any sample−ΔC_T_ for the calibrator].

### Protein extraction and Western blotting

Proteins from tumors were extracted with a buffer solution (20 mM Tris-HCl, pH 7.5, 2 mM ATP, 5 mM MgCl_2_, 1 mM dithiothreitol (DTT), and 5 µL of a protease inhibitor cocktail (Sigma)). Proteins (20 µg/lane) were separated on a 12.5% polyacrylamide gel (a precast SDS gel (Bio-Rad)) and then transferred to a polyvinylidene difluoride membrane (Immobilon, Millipore). Proteins were determined using antibodies against mouse VEGF (1∶500, Novus, USA), mouse Cytochrome c (1∶200, Santa Cruz Biotechnology), mouse caspase 8, 7, 3 (1∶200, Santa Cruz Biotechnology). The antibodies then were stripped off the membrane and re-probed with a specific antibody against β-actin (1∶5000, Novus Biologicals). The intensity was quantified using the Fotodyne Image analysis System (Fotodyne, Hartland, WI, USA) and the TotalLab software (Nonlinear Dynamics, Durham, NC, USA).

### Immunofluorescence assay

Paraffin sections were blocked by a blocking buffer for 1 hour at room temperature and stained with a specific primary antibody at a dilution of 1∶200 for 24 hours. The primary antibody was washed using PBS. The sections then were stained with a specific secondary antibody at a dilution of 1∶100 for 24 hours at room temperature and washed with PBS. The primary antibodies used here are listed as follows: rabbit anti-mouse IL-1β (Novus, USA), rabbit anti-mouse IL-6 (Santa Cruz, USA), rabbit anti-mouse VEGF (Santa Cruz, USA) and rabbit anti-mouse TNF-α (Santa Cruz, USA). The secondary antibody was the FITC-conjugates goat anti-rabbit IgG (Sigma, USA).

### Effects of spleen cells from large-tumor-bearing animals on the activity of CD8^+^ CTL

To determine the suppressive activity found in splenocytes from tumor-bearing animals, we used the modified Winn assay [19]. Briefly, CD8^+^ T cells with the spontaneous anti-tumor lysing activity were first isolated from spleens of mice bearing small line-1 tumors using the BD IMag™ system (BD Biosciences). The resultant cells were estimated by flow cytometry to have >90% CD8^+^ cells (data not shown). These CD8^+^ T lymphocytes were mixed with viable line-1 tumor cells and spleen cells from mice bearing large tumors, which were supplemented with either background or experimental diets 28 days before isolation. To rule out the influence of T cells on antitumor activity, T-cell subsets were depleted from spleen cells by using CD4 and CD8 magnetic beads according to the supplier's protocol (BD Biosciences). The ratios of cells were determined to be 3 purified CD8^+^ splenocytes and 18 spleen cells vs. 1 tumor cell. A mixture of cells (containing 5×10^5^ tumor cells, 15×10^5^ CD8^+^ T cells, and 90×10^5^ spleen cells) was inoculated into the flanks of naive mice. The ratio for CD8^+^ T cells vs. tumor cells has previously been optimized for detecting positive and negative effects [19]. The growth of tumor was measured after 28 days, which was expressed as the mean ± SD of at least three mice per group.

### Statistical Analysis

Data were expressed as means ± SD. Statistical significance was determined by one-way ANOVA through the Bonferroni's multiple comparison test (Prism Graph Pad). Differences were considered statistically significant when *P*<0.05.

## Results

### Cachexia parameters in tumor-bearing mice

In experiment A, mice were divided into two groups, severe and moderate cachexia. The former was defined as those with body-weight loss >10%. Carcass weights in tumor-bearing mice (TB) were significantly less than those in control mice (Con). Final body-weight gain was estimated around 10.2% in normal mice. Body-weight loss therefore can be used to index the tumor-induced cachexia in TB mice. Body-weight loss was estimated about 5.6% in moderately cachectic mice as opposed to 19.1% in severely cachectic mice (data not shown). Loss of muscle and fat masses (e.g., epididymal fat) accounted for the major loss of body weight (Table 1A). Though anorexia usually comes with cancer cachexia, food intakes between the experimental and the control however made no difference (data not shown). Since inflammation and hypoalbuminemia are two main diagnostic indices for cachexia [20], TB mice was subjected to serum albumin analysis. The level of serum albumin was found decreased (Table 1B), where the levels in severe cachectic mice were significantly lower (*P*<0.05, Cancer^Moderate^ vs. Cancer^Severe^) than those of moderate, suggesting hypoalbuminemia is closely associated with weight loss. Serum TNF-α and IL-6 increased otherwise in the group with severe cancer when compared to those of control, whereas IL-1β still kept steady among groups (Table 1B).

**Table 1:** Physiological cachectic and immune parameters (experiment A).

(A) Body, tumor and organ weights						
Treatment	N	BW(g)	TW (g)	CW (g)	eWAT (mg)	mG (mg)
Con	5	28.8±1.9	0.0±0.0	28.8±1.9	362.8±71.8	180.0±36.7
TB^Moderate^	5	28.0±2.1	4.1±1.4	23.9±1.2***	275.7±53.8*	150.3±21.9
TB^Severe^	8	30.8±3.3	8.3±3.1†	22.5±2.8***	116.7±50.5***,†	134.2±29.1**

(B) Cachectic parameters					
Treatment	N	Albumin (g/dl)	TNF-α (pg/ml)	IL-6 (pg/ml)	IL-1β (pg/ml)
Con	5	2.90±0.09	30.6±4.3	14.0±3.4	18.5±6.4
TB^Moderate^	5	2.20±0.05***	69.4±11.7	19.4±5.2	23.4±6.2
TB^Severe^	8	1.98±0.03***,†	98.8±19.2**	27.8±4.8**	28.2±8.0

(C) Immune parameters			
Treatment	N	Thymus (mg)	Spleen (mg)
Con	5	42.8±7.2	154.6±36.0
TB^Moderate^	5	30.3±6.0**	228.0±52.7**
TB^Severe^	8	21.7±3.6***	257.5±50.4***

BW = body weight, TW = Tumor weight, CW = Carcass weight, eWAT = epididymal White Adipose Tissue, mG = muscle Gastrocnemius. Data represent mean ± SD. for n = 15–16 mice/group and were pooled from three independent experiments. Statistical significance was determined by one-way ANOVA.

* p<0.05,

** p<0.01,

*** p<0.001 presents significant differences from Con mice.

† p<0.05 presents significant differences from Cancer^Moderate^ mice.

### Lung cancer up-regulates immunosuppressive cells in mice

**Figure 1:**
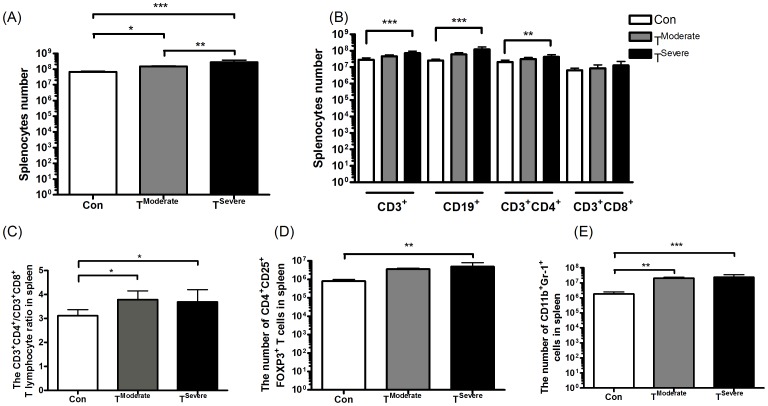
Immunological functions in tumor-bearing mice (experiment A). Mice were s.c. injected with 1×10^5^ line-1 cells. After 35 days, tumors were harvested and splenocytes were extracted and analyzed by flow cytometry. A, Numbers of splenocytes in normal and tumor-bearing mice (n = 15 mice/group pooled from three independent experiments). B, Flow cytometry analysis of CD3^+^ T, CD19^+^ B, CD3^+^CD4^+^ and CD3^+^CD8^+^ T cells in spleen. C, the ratio of CD3^+^CD4^+^ T cells/CD3^+^CD8^+^ T cells in spleen. D and E, the numbers of CD4^+^CD25^+^Fox-p3^+^ Treg (C) and CD11b^+^Gr-1^+^ MDSC (D) cells in spleens of normal and tumor-bearing mice. Data show mean ± SD. for n = 12–13 mice/group and were pooled from three independent experiments. One-way analysis of variance (ANOVA), *p<0.05, **p<0.01, and ***p<0.001 denote levels of significant differences between groups.

Most organ (wet) masses, including kidney, liver, intestine, heart and thymus, were almost unchanged; only slightly decreased in those with cachexia development. In contrast, the sizes of spleens in TB mice were 1.5 times larger than those in control (Table 1C). The volumes of spleens grew perhaps as a result of mass proliferation of splenic cells (Fig. 1A). Previous studies have revealed that the CD4^+^/CD8^+^ ratio for lymphocytes of peripheral blood and peritoneal fluid seems helpful in monitoring disease progression in ovarian or breast cancer patients [21], [22]. Our data agreed with this notion, as the cell ratio of CD3^+^CD4^+^ to CD3^+^CD8^+^ T cells increased in TB mice (*P*<0.05, Fig. 1C)-the number of CD3^+^ in the spleens of TB mice increased (Fig. 1B), but the CD3^+^CD8^+^T cells did not increase (Fig. 1C). We further assessed the CD4^+^CD25^+^Fox-p3^+^ Tregs in spleens of TB mice. As shown in Fig. 1D, the number of CD4^+^CD25^+^Fox-p3^+^ Treg significantly increased (*P*<0.05). On the other hand, MDSC was recently identified to be CD11b^+^Gr-1^+^, which was found significantly increased in a number of cancers, including head-neck, pancreatic, HCC, renal, and breast cancers [23]. The average number of CD11b^+^Gr-1^+^ cells in the spleens of naive BALB/c mice was measured to be 1.8±0.2×10^6^ (Fig. 1E). Corresponding cells in TB mice also markedly increased, in which the CD11b^+^Gr-1^+^ cells accounted for 13.9% of the spleen cells, equivalent to 20.2±1.7×10^6^ CD11b^+^Gr-1^+^ cells in a moderate tumor (4.1 g), an 11-fold increase (**P<0.01 versus non-tumor-bearing animals) (Fig. 1D). Fig. 1 demonstrates that splenomegaly in TB mice is positively correlated to the total numbers of CD4^+^CD25^+^Fox-p3^+^ Tregs plus CD11b^+^Gr-1^+^ cells.

### Fish oil and selenium yeast inhibit tumor growth and attenuate cachectic symptoms

All physiological parameters (i.e. weights of liver, kidney, spleen, and uterine) in non-tumor-bearing mice (Con) (receiving normal experimental diet) did not display apparent side effects. Addition of fish oil (TB-fo) or selenium yeast (TB-se) in the background diet also did not alter body compositions (Table 2A). However, addition of fish oil plus selenium yeast (TB+fo+se) significantly increased the delta weights of carcass, fat (epididymal) and muscle (mTA) when compared to those in TB-Con (Table 2A). On the other hand, the tumor weights in the mice treated with either fish oil or selenium yeast made no big difference from those in the mice treated with vehicle control. Interestingly, the tumor weights in the mice treated with both (fo+se) were ∼58% less than those in control, suggesting the combination of fish oil and selenium yeast acts synergistically in suppression of tumor growth (Table 2A).

**Table 2:** Effect of the specific nutritional combination (SNC) on physiological cachectic parameters (experiment B).

(A) Physiological cachexia parameters							
Treatment	N	BW (g)	TW (g)	CW (g)	Delta CW	eWAT (g)	mG (g)
Con	5	27.8±1.8	0.0±0.0***	27.8±1.8	11.8±5.0***	387.8±58.5***	204.0±33.6***
TB-Con	6	28.5±1.1	5.7±2.2	22.7±1.8	−10.0±6.0	112.9±63.2	131.4±32.4
TB+fo	6	27.7±2.1	3.7±1.6	24.0±1.0	−4.5±2.8	188.3±50.0	171.7±24.8
TB+se	6	27.5±2.1	4.2±2.4	23.3±1.5	−7.2±5.7	176.7±58.2	151.5±17.1
TB+fo+se	10	27.7±1.8	2.4±1.9*	25.3±1.9	0.3±5.2**	235.7±64.3**	182.9±18.0**

(B) Cachectic parameters					
Treatment	N	Albumin (g/dl)	TNF-α (pg/ml)	IL-6 (pg/ml)	IL-1β (pg/ml)
Con	5	3.02±0.08***	33.3±3.7***	13.1±4.7	20.3±5.3
TB-Con	6	2.40±0.08	68.8±3.8	16.4±5.3	25.3±6.2
TB+fo	6	2.51±0.05	54.0±3.5**	14.0±4.9	23.1±6.6
TB+se	6	2.55±0.07	60.8±4.3*	15.4±4.7	21.6±7.8
TB+fo+se	10	2.64±0.10**	49.7±3.8***	13.0±4.6	21.8±5.9

(C) Food intake in time (gram per day)						
Treatment	N	Day 1	Day 10	Day 20	Day 30	Day 41
Con	5	4.8	4.2	4.6	5.1	4.8
TB-Con	6	4.9	4.3	4.0	4.5	3.8
TB+fo	6	4.6	4.0	4.3	4.8	4.2
TB+se	6	4.7	3.9	4.4	4.8	4.3
TB+fo+se	10	4.8	4.5	4.6	4.4	4.5

Con = mice receiving control diet, TB-Con = tumor-bearing mice receiving control diet, fo = fish oil, se = selenium yeast, BW = body weight, TW = Tumor weight, CW = Carcass weight, delta CW = CW day 42 minus CW day 0, eWAT = epididymal White Adipose Tissue, mG = muscle Gastrocnemius, Data represent mean ± SD. for n = 15–16 mice/group and were pooled from three independent experiments. Statistical significance was determined by one-way ANOVA.

* p<0.05,

** p<0.01,

*** p<0.001 presents significant differences from TB-Con mice.

In contrast to TB-Con, the diet with fish oil and selenium yeast improved hypoalbuminemia in TB mice (Table 2B). Addition of either nutrient in diet reduced serum TNF-α; this effect was fortified by addition of both nutrients (Table 2B). One should note that there was no difference among groups in terms of food intakes (Table 2C), and that the tumor-induced cachectic symptoms were more severe in experiment A than those in B. Since TB mice received the background diet in the experiment B, these improvements were ascribed to as a result of addition of the nutrients in the diet.

### Fish oil and selenium yeast down-regulate immunosuppressive cell population

**Figure 2:**
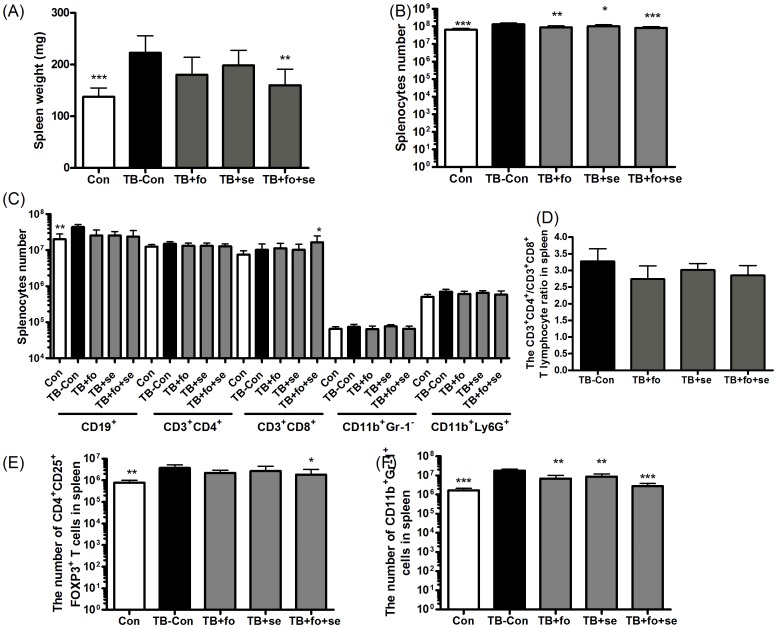
Effects of single or combined nutrients on tumor induced immune suppressions in spleens (experiment B). Mice received single or combined nutrients for 42 days after tumor inoculation. The whole spleens were homogenized and analyzed for expressions of cell surface markers and intracellular markers by flow cytometry. A and B, Effects of single or combined nutrients on spleen weights (A) and splenocytes numbers (B). C, Flow cytometry analyses of CD19^+^ B, CD3^+^CD4^+^ T, CD3^+^CD8^+^ T cells, CD11b^+^Gr-1^−^ macrophages and CD11b^+^Ly6G^+^ monocytes in spleens. D, The ratios of CD3^+^CD4^+^ T cells/CD3^+^CD8^+^ T cells in spleens. E and F, Cell numbers of CD4^+^CD25^+^Fox-p3^+^ Treg (E) and CD11b^+^Gr-1^+^ MDSC (F) in spleens of normal and tumor-bearing mice. Data show mean ± SD. for n = 14–15 mice/group pooled from three independent experiments. *p<0.05, **p<0.01, and ***p<0.001 represent levels of significant differences from TB-Con mice.

To examine whether the anti-tumor activity of the nutrients is through inhibiting tumor growth, the indicative immune parameters in the spleens or peripheral lymph nodes (PLN) of TB mice were measured (Fig. 2 and S2). First, we found spleens were markedly prevented from enlargement (Fig. 2A), in line with that the number of splenic cells decreased in the group receiving the combination of fish oil and selenium yeast (Fig. 2B). Second, we examined populations of suppressive immune cells. The cell numbers of CD3^+^CD4^+^, CD3^+^CD8^+^, CD4^+^CD25^+^Fox-p3^+^, and CD11b^+^Gr-1^+^ in the spleens of TB-Con were determined using flow cytometry. As shown in Fig. 1 and 2, the cell populations of CD4^+^CD25^+^Fox-p3^+^ Tregs and CD11b^+^Gr-1^+^ MDSC, and the cell ratio of CD3^+^CD4^+^/CD3^+^CD8^+^ are relatively higher in the TB-Con mice in comparison to those in Con mice. One may speculate that the background diet may have some degree of anti-tumor activity, but the fact is the populations of CD4^+^CD25^+^Fox-p3^+^ Tregs and CD11b^+^Gr-1^+^ MDSCs in TB mice are much higher than those in TB-Con mice (Fig. 2E). The cell numbers of MDSC decreased in the spleens and PLN of the group orally administrated with either fish oil or selenium yeast, while they were further lowered down in the group supplemented with the both nutrients (Fig. 2F and S2). By contrast, the overall number of CD19^+^ B, CD3^+^CD4^+^ T, CD11b^+^Gr-1^−^ macrophage, CD11b^+^Ly6G^+^ monocyte and the ratio of CD3^+^CD4^+^/CD3^+^CD8^+^ did not fluctuate in spleen or PLN in the group receiving the combined nutrients (TB+fo+se) (Fig. 2C, D and S2). This result suggests that the combination of fish oil and selenium yeast may be specific to Tregs and MDSCs.

### Combination of fish oil and selenium yeast down-regulates tumor-associated MDSCs suppressive mediators

**Figure 3:**
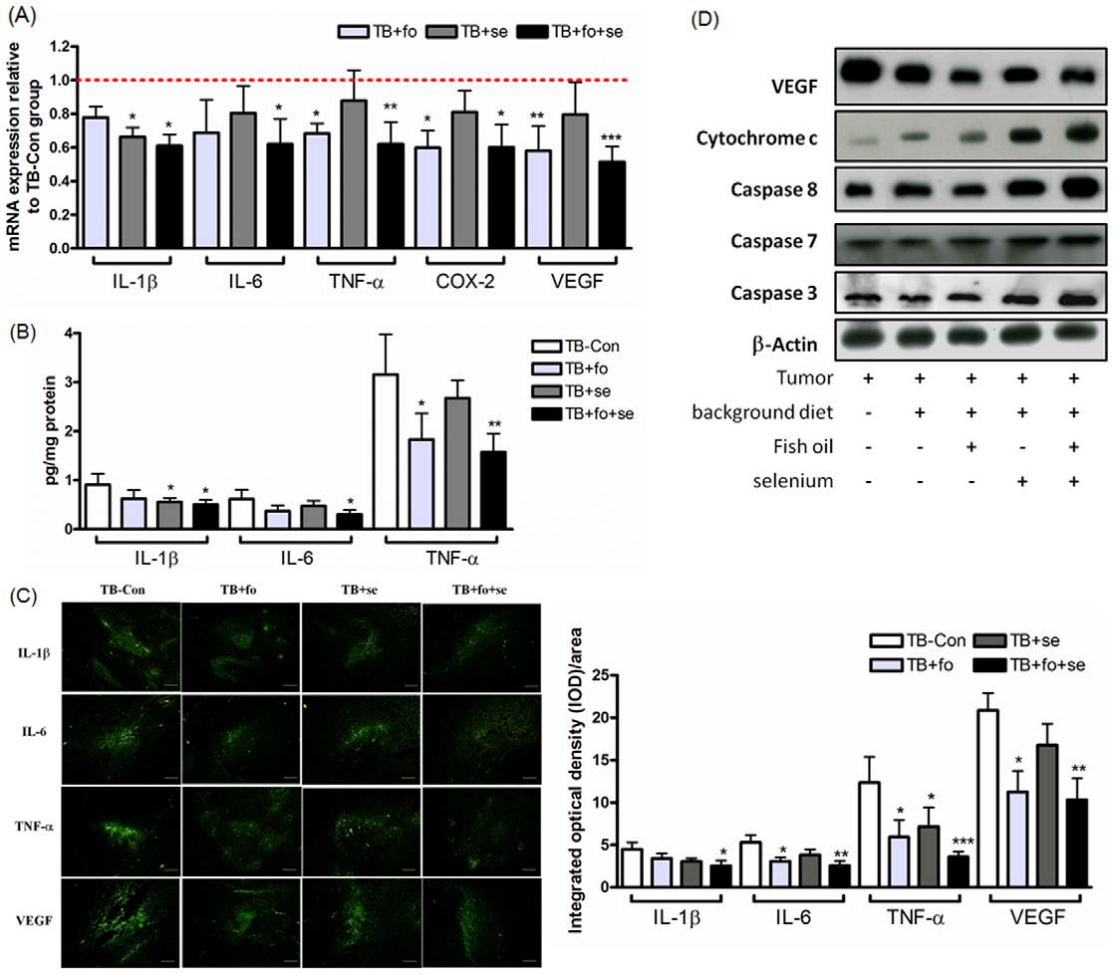
Combination of nutrients modulates tumor-induced cachexia and immunosuppression factors (experiment B). A, mRNA levels of cachexic and immunosuppression factors in tumors with treatment of the nutrients combination. Values are means of fluorescence signals expressed as percentages (normalized to GAPDH mRNA). Data show mean ± SD. for n = 10–12 mice/group pooled from three independent experiments. B, Tumors (n = 10 mice/group pooled from three independent experiments) were homogenized for determining proinflammatory mediators by ELISA. C, Immunofluorescence analysis for untreated (TB-Con) and treated lung tumors stained with IL-1β, IL-6, TNF-α and VEGF. Images are shown at ×100 magnification. Immunostained expression levels of immunosuppression proteins that were quantitated using Image Pro Plus 6.0 software (Media Cybernetics Inc., New York, USA). Data show mean ± SD. for n = 7–8 mice/group pooled from three independent experiments. D, VEGF and apoptosis-associated proteins in tumors from above that were Western blotted using appropriate specific antibodies. Tumor(+)Background diet(−)Fish oil(−)Selenium(−) indicates “in vitro cultured tumor cells”. Data show n = 10–12 mice/group, and mean ± SD. is representative of results from three independent experiments. *p<0.05, **p<0.01, and ***p<0.001 stand for levels of significant differences from TB-Con mice.

It has been known that the accumulation and activation of MDSCs are driven by a multitude of factors, many of which provoke chronic inflammation [24]. Whether the fish oil and/or selenium yeast mediated-tumor inhibition results from reduction of immunosuppressive cells, the modulation of proinflammatory mediators was examined. As shown in Fig. 3A and B, the levels of TNF-α and COX-2 significantly decreased in the TB mice treated with fish oil alone (TB+fo), whereas IL-1β decreased in the mice treated with selenium yeast (TB+se). The expressions of IL-1β, IL-6 and TNF-α were also analyzed using fluorescence microscopy (Fig. 3C). The expressions of IL-6 andTNF-α dropped 1.7 and 2.1 folds respectively in comparison to those in the TB-Con mice.

It has been well documented that chronic inflammation promotes tumor onset and development by angiogenesis, anti-apoptosis, and stimulates tumor cell proliferation and metastasis [24]. Previous studies have also demonstrated that VEGF or its receptors are up-regulated in many human cancers [25]. In this study, the mRNA and protein levels of VEGF significantly reduced in the TB mice treated with fish oil (Fig. 3). Selenium yeast did not affect the expression of VEGF in the TB tumors. On the contrary, it showed a strong apoptotic effect (Fig. 3D). We suggested the selenium yeast-induced apoptosis is through activating the caspase cascades of mitochondria and the Fas/FasL pathway.

### Fish oil and selenium yeast attenuate immunosuppression on CD8^+^ CTL

**Figure 4:**
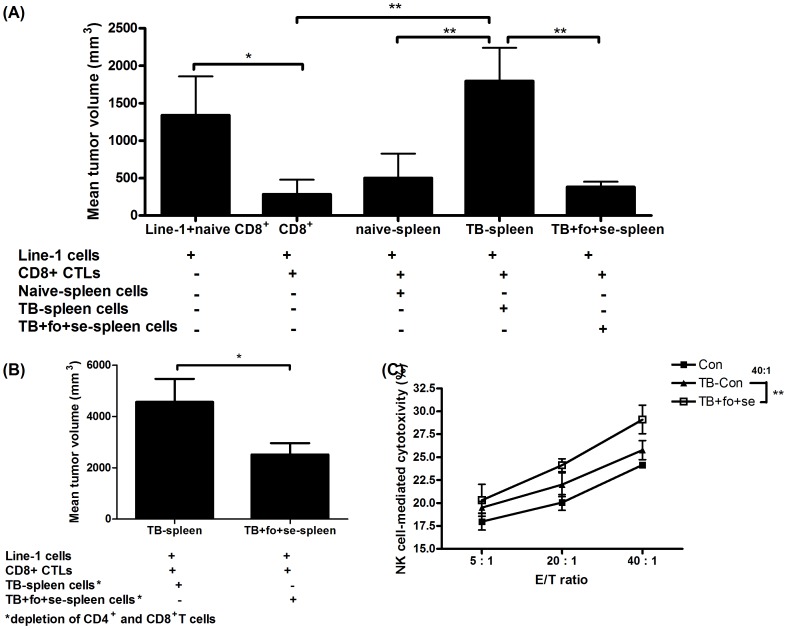
Dietary supplementation with combined fish oil and selenium yeast reduces immunosuppressive activity of spleen cells in tumor-bearing animals on CD8^+^ CTL and NK cells activities. A, Winn assays were used to quantify inhibitory effects of splenocytes from tumor-bearing mice (n = 9–10 mice/group from two independent experiments) on endogenous CD8^+^ T cell activity. B, Dietary supplementation with fish oil and selenium yeast was shown to enhance the CD8^+^ CTL activity even after depletion of CD4− and CD8− specific T cells in splenocytes. Data show n = 9–10 mice/group, and mean ± SD. is representative of results from two independent experiments. C, Tumors in mice were intervened with or without addition of fish oil and selenium yeast. After sacrifice, splenocytes from BALB/cByJ mice were used as the effector cells to test the protective effect of experimental diets for the NK-associated cytotoxicity against target cells (line-1). Data show mean ± SD. of n = 7–8 mice/group and are representative of results from three independent experiments. *p<0.05, and **p<0.01 denote levels of significant differences between groups.

Spleen CD11b^+^Gr-1^+^ cells in some TB mice were reported able to suppress CD8^+^ T cell activation [26]. Since the immunosuppressive cell populations in spleens can be reduced by fish oil and selenium, we considered that equal cell numbers of splenocytes from TB-Con mice should theoretically be more suppressive than corresponding splenocytes from the TB+fo+se mice. This hypothesis was tested using the modified Winn assay, whereby the activities of anti-tumor CD8^+^ cytotoxic T lymphocyte (CTL) were compared with the presence of splenocytes from the treated or non-treated mice [27]. One should also note that the mice bearing line-1 tumors would spontaneously develop CD8^+^ T cells in spleens that have a specific and potent tumor lytic activity. As expected, tumor growth was markedly inhibited after 28 days in the group injected with splenocytes from the TB+fo+se mice as opposed to that with the naive splenic CD8^+^ T group (*P<0.05, Fig. 4A). To determine the suppressive effect of CD11b^+^Gr-1^+^ cells on the CD8^+^ CTL activity, line-1 cells were mixed with active CD8^+^ CTLs along with spleen cells from naive mice or TB mice, or the TB mice treated with fish oil and selenium yeast for 28 days. As shown in Fig. 4A, when the given ratio of splenocytes from native or tumor-bearing mice were mixed with CD8^+^ T cells, the tumor growth inhibitory effect of CD8^+^ CTLs faded away (**P<0.01, Fig. 4A), wherein tumor cells grew as fast as line-1 cells implanted alone. However, when splenocytes from TB mice receiving the combination of fish oil and selenium yeast were mixed with the line-1 cells plus CD8^+^ CTLs, the CD8^+^ CTLs retained the growth inhibitory activity (**P<0.01, Fig. 4A). To rule out a possible influence of T cells in the spleen of the TB+fo+se mice on activation of anti-tumor activity, CD4^+^ and CD8^+^ T cells in splenocytes were subjected to depletion by magnetic activated cell sorting, whereby the TB mice with depletion of the T-cell subset and with treatment of fish oil and selenium yeast (TB+fo+se) indeed presented stronger anti-tumor activity (*P<0.05, Fig. 4B).

Next, we evaluated the effect of the combined nutrients in the TB mice (TB+fo+se) on the cytotoxicity of endogenous NK cells versus target cells line-1 and YAC-1. Two fluorescent dyes, DIOC18 and PI, were used to stain cells. An E/T ratio of 40/1 reached the maximal efficacy on NK cytotoxicity in the TB+fo+se group, wherein around 28% of the line-1 and YAC-1 cells were stained with PI (**P<0.01, Fig. 4C and 4S) when compared to those in the TB-Con group. As a result, the activities of CD8^+^ CTL and NK cells were inhibited in the TB mice; however, the activities can be restored to a significant extent when the cell numbers of CD11b^+^Gr-1^+^ were lowered down by supplementing the TB mice with the combination of fish oil and selenium yeast.

## Discussion

Cachexia is an ensemble of illness, manifestations with loss of lean body and fat masses, metabolic abnormality, inflammation, and impaired immune function, anorexia, etc. Some animal models have been developed for the cancer cachexia study. For example, C-26 and MAC16 colon adenocarcinoma tumor-bearing mice were the most known models, while the major disadvantage in these models is the developed tumor masses only 3% of body weight [28]. Lung cancer is the cancer that is most often complicated with cachexia [29]; no animal model was available for exploring the lung cancer-induced cachexia. In this study, a purpose-oriented mice model with murine lung carcinoma (line-1) that develops progressive cachexia was validated, in which carcass, muscle and fat weights (Table 1A, experiment A) grow significantly under a normal food-intake condition.

Weight loss, lean body mass decrease, and hypermetabolism are highly correlated to both the elevated levels of proinflammatory cytokines, such as IL-1β, IL-6 and TNF-α and the decreased level of albumin in lung cancer patients [30]. EPA and DHA that inhibit production of IL-1β and TNF-α of human monocytes have been demonstrated in cell-culture studies [31]. Endres et al. [32] evaluated the effect of addition of fish-oil into a normal Western diet in healthy volunteers, showing that productions of IL-1β, IL-1α and TNF can restore to the pre-supplement level in the end of supplementation. Moreover, the streptozotocin-induced diabetic mice treated with selenium resulted in suppressions of proinflammatory cytokines IL-1β and TNF-α [33]. The level of IL-1β was not significantly altered in this study, while the serum levels of TNF-α and IL-6 did increase (Table 1B, experiment A). This discrepancy may be ascribed to the sensitivity of the method used, a short half-life and/or diurnal fluctuations of IL-1β. In experiments B, the combination of fish oil and selenium yeast demonstrated a significant effect on the body index and hypoalbuminemia. Additionally, the level of tumor-induced TNF-α decreased in the group treated with the combined nutrients, which pretty much agrees with previous investigations. One should note that the tumor induced cachectic symptoms were more serious in experiment A than in B, because the tumor-bearing mice in the category B experiment received background diets only, suggesting the background diet may have certain degree of the anti-tumor effect.

That spleens underwent enlargement in the TB mice in fact is similar to those from other model studies [34]-the spleen enlargement is result of the immune system in response to tumors. The line-1 cells grow faster in mice in spite of provocation of strong immune responses, suggesting anti-tumor activity is inhibited. It has been well documented that MDSCs and Tregs accumulate considerably in both tumor and secondary lymphoid tissues [4], [5]. In our study both Tregs and MDSCs significantly up-regulated in TB mice are well correlated with tumor burdens. Systemic accumulation of MDSCs and Tregs is often associated with poor prognosis and immunosuppression. This study agrees with this notion-natural killer (NK) and CD8^+^ T cells are suppressed in spleens of animals bearing tumors. On the contrary, CD8^+^ T cells and activated NK cells significantly increased, likely as a result of decrease of MDSCs and Tregs upon treating animals with the combined nutrients (Fig. 4). This outcome strongly suggests that the combination of fish oil and selenium yeast has an *in vivo* immunomodulatory effect on lung carcinoma.

MDSCs accumulation and activation have been known deriving from multiple factors, many of which are related with chronic inflammation. For example, the inflammation-associated VEGF was reported in association with accumulation of MDSCs [35]. In this study the levels of TNF-α, IL-6, COX-2 and VEGF in tumor microenvironment were reduced by virtue of addition of fish oil (Fig. 3). This result is well in line with that omega-3 polyunsaturated fatty acids (PUFAs) down-regulate COX-2, TNF-α and VEGF in microphages or tumors [36]. Thereby, it is plausible to propose that the reduction of immunosuppressive cell populations mediated by fish oil is likely through attenuating expressions of inflammatory cytokines and VEGF. Besides, peanut oil (which is rich in omega 6) was used to compare with fish oil; the latter was proved to have better anti-tumor and anti-cachexia effects (Fig. S3).

Selenium required for proper functionality of macrophages and T lymphocytes should play an important role in anti-inflammation [37]. Zeng et al. [45] similarly reported selenium supplementation can enhance caspase-dependent apoptosis in tumors. Administration of selenite has been reported to increase IL-1β mRNA expressions in pancreas in diabetic model mice [38]. On the other hand, IL-1β was reported able to promote angiogenesis [39] as well as enhance accumulation of splenic MDSCs [40] by suppressing NK cell development and functioning [41]. IL-1β-converting enzyme (ICE), known as caspase-1, is a protease in converting inactive IL-1β to active [42], and is known to inhibit activation of apoptosis [43]. Selenium recently was reported capable of inhibiting caspase-1 activation [44], so as to suppress tumor angiogenesis [45]. We found that the treatment of selenium yeast truly can neutralize expression of IL-1β (Fig. 3). Therefore, selenium may limit MDSCs accumulation through mediating the level of IL-1β. The NK cell activity in the splenocytes from the TB mice treated with fish oil and selenium yeast was similar to those from normal mice, likely through reducing the number of myeloid suppressor cells.

In summary, we have established an immunocompetent lung carcinoma mouse model that facsimiles human lung cancer. The line-1 tumor cell-induced cancer cachexia presents typical characteristic symptoms, including muscle wasting, chronic inflammation, disturbed immune function, but no anorexia. This model enabled us to gain further insights into the immune responses of lung cancer in the presence/absence of fish oil and/or selenium yeast. Experimental results concluded that fish oil and selenium yeast together are able to reduce the number of suppressor cells in the spleens of animals bearing tumors but have no influences on B cells, monocytes, and macrophages. The significant decline of the suppressor cells is likely as a result of increase of anti-tumor CD8^+^ T cells and activated NK cells.

## Supporting Information

Figure S1 **Protocols for lung cancer-induced cachexia in mice and nutritional supplementation in tumor-bearing mice.** A, In the experiment A, BALB/cByJ mice (6–7 weeks) were inoculated subcutaneously (s.c.) with a homogenate of line-1 tumor cells (1×10^5^) on day 0. The control group was injected with 0.1 ml of sterile saline solution. Mice are sacrificed and analyzed 35 days after tumor inoculation. B, In the experiment B, the supplements were administered orally with experiment diet (1 g/mice/day) after 5×10^4^ tumor inoculation, while the control mice received background diet (1 g/mice/day). Mice are kept on experiment diets until sacrifice and analysis at 42 days after tumor inoculation.
(TIF)

Figure S2 **Effects of the combined nutrients on tumor induced immune suppressions in peripheral lymph nodes (experiment B).** The combined nutrients were fed to mice for 36 days after tumor inoculation. PLN were homogenized and analyzed for CD19^+^ B, CD3^+^CD4^+^ T, CD3^+^CD8^+^ T cells, CD4^+^CD25^+^Fox-p3^+^ Tregs and CD11b^+^Gr-1^+^ MDSC in normal and tumor-bearing mice. Data show mean ± SD. of n = 7–8 mice/group and are representative of results from two independent experiments. *p<0.05, **p<0.01, and ***p<0.001 represent levels of significant differences among TB-Con mice.
(TIF)

Figure S3 **Effects of fish oil and peanut oil on tumor growth.** Mice bearing line-1 tumors were treated p.o. with 150 mg/day of fish oil or peanut oil, and sacrificed by CO_2_ inhalation method on day 42. Data show mean ± SD. of n = 10–12 mice/group and are representative of results from three independent experiments.
(TIF)

Figure S4 **Effect of the combined nutrients on NK cytotoxicity in the spleen of BALB/c mice.** Tumors in mice (n = 6–7 mice/group) were intervened with or without addition of fish oil and selenium yeast. After sacrifice, splenocytes from BALB/cByJ mice were used as the effector cells to test the protective effect of experimental diets for the NK-associated cytotoxicity against target cells (YAC-1). All values are means ± SD. **p<0.01 denote levels of significant differences between groups.
(TIF)

## References

[1] Van Cutsem E, Arends J (2005) The causes and consequences of cancer-associated malnutrition. Eur J Oncol Nurs 9 Suppl 2: S51–63. 16437758 10.1016/j.ejon.2005.09.007

[2] Evans C, Dalgleish AG, Kumar D (2006) Review article: immune suppression and colorectal cancer. Aliment Pharmacol Ther 24: 1163–1177. 17014575 10.1111/j.1365-2036.2006.03075.x

[3] Herber DL, Nagaraj S, Djeu JY, Gabrilovich DI (2007) Mechanism and therapeutic reversal of immune suppression in cancer. Cancer Res 67: 5067–5069. 17545581 10.1158/0008-5472.CAN-07-0897 PMC1976287

[4] Beyer M, Schultze JL (2006) Regulatory T cells in cancer. Blood 108: 804–811. 16861339 10.1182/blood-2006-02-002774

[5] Ueha S, Shand FH, Matsushima K (2011) Myeloid cell population dynamics in healthy and tumor-bearing mice. International immunopharmacology 11: 783–788. 21406269 10.1016/j.intimp.2011.03.003

[6] Li H, Han Y, Guo Q, Zhang M, Cao X (2009) Cancer-expanded myeloid-derived suppressor cells induce anergy of NK cells through membrane-bound TGF-beta 1. J Immunol 182: 240–249. 19109155 10.4049/jimmunol.182.1.240

[7] Cohen AC, Nadeau KC, Tu W, Hwa V, Dionis K, et al (2006) Cutting edge: Decreased accumulation and regulatory function of CD4+ CD25(high) T cells in human STAT5b deficiency. J Immunol 177: 2770–2774. 16920911 10.4049/jimmunol.177.5.2770

[8] Sakaguchi S (2004) Naturally arising CD4+ regulatory t cells for immunologic self-tolerance and negative control of immune responses. Annual review of immunology 22: 531–562. 10.1146/annurev.immunol.21.120601.141122 15032588

[9] van der Meij BS, Langius JA, Smit EF, Spreeuwenberg MD, von Blomberg BM, et al (2010) Oral nutritional supplements containing (n-3) polyunsaturated fatty acids affect the nutritional status of patients with stage III non-small cell lung cancer during multimodality treatment. J Nutr 140: 1774–1780. 20739445 10.3945/jn.110.121202

[10] Takagi K, Yamamori H, Furukawa K, Miyazaki M, Tashiro T (2001) Perioperative supplementation of EPA reduces immunosuppression induced by postoperative chemoradiation therapy in patients with esophageal cancer. Nutrition 17: 478–479. 11399408 10.1016/s0899-9007(01)00557-3

[11] Furukawa K, Tashiro T, Yamamori H, Takagi K, Morishima Y, et al (1999) Effects of soybean oil emulsion and eicosapentaenoic acid on stress response and immune function after a severely stressful operation. Ann Surg 229: 255–261. 10024108 10.1097/00000658-199902000-00014 PMC1191639

[12] McCarthy DO (2003) Rethinking nutritional support for persons with cancer cachexia. Biol Res Nurs 5: 3–17. 12886666 10.1177/1099800403005001001

[13] Roth MJ, Qiao YL, Abnet CC, Zhang YH, Dawsey SM, et al (2006) Cellular immune response is not associated with incident cancer or total mortality: a prospective follow-up. Eur J Cancer Prev 15: 548–550. 17106336 10.1097/01.cej.0000220632.93104.2d

[14] Kiremidjian-Schumacher L, Roy M (2001) Effect of selenium on the immunocompetence of patients with head and neck cancer and on adoptive immunotherapy of early and established lesions. Biofactors 14: 161–168. 11568453 10.1002/biof.5520140121

[15] Faber J, Vos AP, Kegler D, Argiles J, Laviano A, et al (2009) Impaired immune function: an early marker for cancer cachexia. Oncol Rep 22: 1403–1406. 19885593 10.3892/or_00000581

[16] Burns CP, Halabi S, Clamon G, Kaplan E, Hohl RJ, et al (2004) Phase II study of high-dose fish oil capsules for patients with cancer-related cachexia. Cancer 101: 370–378. 15241836 10.1002/cncr.20362

[17] Yuhas JM, Pazmino NH, Proctor JO, Toya RE (1974) A direct relationship between immune competence and the subcutaneous growth rate of a malignant murine lung tumor. Cancer Res 34: 722–728. 4360837

[18] Dussault AA, Pouliot M (2006) Rapid and simple comparison of messenger RNA levels using real-time PCR. Biol Proced Online 8: 1–10. 16446781 10.1251/bpo114 PMC1352391

[19] Xia S, Sha H, Yang L, Ji Y, Ostrand-Rosenberg S, et al (2011) Gr-1+ CD11b+ myeloid-derived suppressor cells suppress inflammation and promote insulin sensitivity in obesity. J Biol Chem 10.1074/jbc.M111.237123 PMC3123122 21592961

[20] Evans WJ, Morley JE, Argiles J, Bales C, Baracos V, et al (2008) Cachexia: a new definition. Clin Nutr 27: 793–799. 18718696 10.1016/j.clnu.2008.06.013

[21] Sevcikova L, Hunakova L, Chorvath B, Turzova M, Boljesikova E (1992) T-lymphocyte subsets (CD4/CD8 ratio) in breast cancer patients. Neoplasma 39: 219–222. 1436231

[22] Markowska J, Lacki JK, Jaroszewski J, Wiktorowicz K (1995) The usefulness of CD4/CD8 ratio evaluation in monitoring of ovarian cancer patients. European journal of gynaecological oncology 16: 54–58. 7744118

[23] Youn JI, Nagaraj S, Collazo M, Gabrilovich DI (2008) Subsets of myeloid-derived suppressor cells in tumor-bearing mice. J Immunol 181: 5791–5802. 18832739 10.4049/jimmunol.181.8.5791 PMC2575748

[24] Baniyash M (2004) TCR zeta-chain downregulation: curtailing an excessive inflammatory immune response. Nat Rev Immunol 4: 675–687. 15343367 10.1038/nri1434

[25] Takekoshi K, Isobe K, Yashiro T, Hara H, Ishii K, et al (2004) Expression of vascular endothelial growth factor (VEGF) and its cognate receptors in human pheochromocytomas. Life Sci 74: 863–871. 14659975 10.1016/j.lfs.2003.07.036

[26] Kusmartsev S, Nefedova Y, Yoder D, Gabrilovich DI (2004) Antigen-specific inhibition of CD8+ T cell response by immature myeloid cells in cancer is mediated by reactive oxygen species. J Immunol 172: 989–999. 14707072 10.4049/jimmunol.172.2.989

[27] DeLong P, Tanaka T, Kruklitis R, Henry AC, Kapoor V, et al (2003) Use of cyclooxygenase-2 inhibition to enhance the efficacy of immunotherapy. Cancer Res 63: 7845–7852. 14633712

[28] Beck SA, Tisdale MJ (1987) Production of lipolytic and proteolytic factors by a murine tumor-producing cachexia in the host. Cancer Res 47: 5919–5923. 3311359

[29] Ross PJ, Ashley S, Norton A, Priest K, Waters JS, et al (2004) Do patients with weight loss have a worse outcome when undergoing chemotherapy for lung cancers? Br J Cancer 90: 1905–1911. 15138470 10.1038/sj.bjc.6601781 PMC2409471

[30] Martin F, Santolaria F, Batista N, Milena A, Gonzalez-Reimers E, et al (1999) Cytokine levels (IL-6 and IFN-gamma), acute phase response and nutritional status as prognostic factors in lung cancer. Cytokine 11: 80–86. 10080883 10.1006/cyto.1998.0398

[31] Baldie G, Kaimakamis D, Rotondo D (1993) Fatty acid modulation of cytokine release from human monocytic cells. Biochimica et biophysica acta 1179: 125–133. 8218354 10.1016/0167-4889(93)90133-a

[32] Endres S, Ghorbani R, Kelley VE, Georgilis K, Lonnemann G, et al (1989) The effect of dietary supplementation with n-3 polyunsaturated fatty acids on the synthesis of interleukin-1 and tumor necrosis factor by mononuclear cells. The New England journal of medicine 320: 265–271. 2783477 10.1056/NEJM198902023200501

[33] Zeng J, Zhou J, Huang K (2009) Effect of selenium on pancreatic proinflammatory cytokines in streptozotocin-induced diabetic mice. The Journal of nutritional biochemistry 20: 530–536. 18789669 10.1016/j.jnutbio.2008.05.012

[34] DuPre SA, Hunter KW Jr (2007) Murine mammary carcinoma 4T1 induces a leukemoid reaction with splenomegaly: association with tumor-derived growth factors. Exp Mol Pathol 82: 12–24. 16919266 10.1016/j.yexmp.2006.06.007

[35] Almand B, Clark JI, Nikitina E, van Beynen J, English NR, et al (2001) Increased production of immature myeloid cells in cancer patients: a mechanism of immunosuppression in cancer. Journal of immunology 166: 678–689. 10.4049/jimmunol.166.1.678 11123353

[36] Ren J, Chung SH (2007) Anti-inflammatory effect of alpha-linolenic acid and its mode of action through the inhibition of nitric oxide production and inducible nitric oxide synthase gene expression via NF-kappaB and mitogen-activated protein kinase pathways. J Agric Food Chem 55: 5073–5080. 17542608 10.1021/jf0702693

[37] Hoffmann PR (2007) Mechanisms by which selenium influences immune responses. Arch Immunol Ther Exp (Warsz) 55: 289–297. 18219759 10.1007/s00005-007-0036-4

[38] Zeng J, Zhou J, Huang K (2009) Effect of selenium on pancreatic proinflammatory cytokines in streptozotocin-induced diabetic mice. J Nutr Biochem 20: 530–536. 18789669 10.1016/j.jnutbio.2008.05.012

[39] Shchors K, Shchors E, Rostker F, Lawlor ER, Brown-Swigart L, et al (2006) The Myc-dependent angiogenic switch in tumors is mediated by interleukin 1beta. Genes Dev 20: 2527–2538. 16980582 10.1101/gad.1455706 PMC1578676

[40] Song X, Krelin Y, Dvorkin T, Bjorkdahl O, Segal S, et al (2005) CD11b+/Gr-1+ immature myeloid cells mediate suppression of T cells in mice bearing tumors of IL-1beta-secreting cells. Journal of immunology 175: 8200–8208. 10.4049/jimmunol.175.12.8200 16339559

[41] Elkabets M, Ribeiro VS, Dinarello CA, Ostrand-Rosenberg S, Di Santo JP, et al (2010) IL-1beta regulates a novel myeloid-derived suppressor cell subset that impairs NK cell development and function. European journal of immunology 40: 3347–3357. 21110318 10.1002/eji.201041037 PMC3373225

[42] Dinarello CA (2011) Interleukin-1 in the pathogenesis and treatment of inflammatory diseases. Blood 117: 3720–3732. 21304099 10.1182/blood-2010-07-273417 PMC3083294

[43] Tatsuta T, Cheng J, Mountz JD (1996) Intracellular IL-1beta is an inhibitor of Fas-mediated apoptosis. Journal of immunology 157: 3949–3957. 8892627

[44] Moon PD, Kim HM (2011) The suppression of thymic stromal lymphopoietin expression by selenium. Amino Acids 10.1007/s00726-011-1156-z 22086213

[45] Ip C (1998) Lessons from basic research in selenium and cancer prevention. J Nutr 128: 1845–1854. 9808633 10.1093/jn/128.11.1845

