# Supplementary figures and images for: Reduction of Splenic Immunosuppressive Cells and Enhancement of Anti-Tumor Immunity by Synergy of Fish Oil and Selenium Yeast
Source: PLoS One. 2013 Jan 22;8(1):e52912. doi: 10.1371/journal.pone.0052912 (PMC3551929; doi:10.1371/journal.pone.0052912)

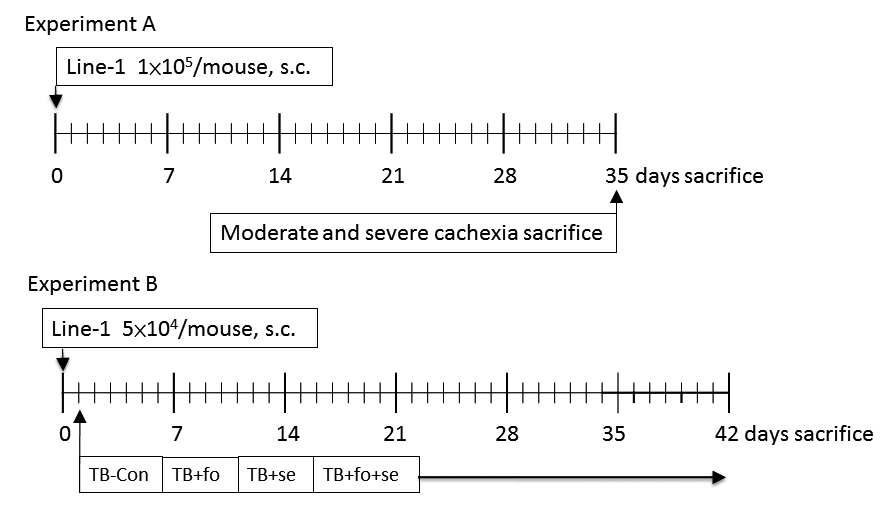

Supplement: Figure S1 — Protocols for lung cancer-induced cachexia in mice and nutritional supplementation in tumor-bearing mice. A, In the experiment A, BALB/cByJ mice (6–7 weeks) were inoculated subcutaneously (s.c.) with a homogenate of line-1 tumor cells (1×10^5^) on day 0. The control group was injected with 0.1 ml of sterile saline solution. Mice are sacrificed and analyzed 35 days after tumor inoculation. B, In the experiment B, the supplements were administered orally with experiment diet (1 g/mice/day) after 5×10^4^ tumor inoculation, while the control mice received background diet (1 g/mice/day). Mice are kept on experiment diets until sacrifice and analysis at 42 days after tumor inoculation. (TIF) [file pone.0052912.s001.tif]

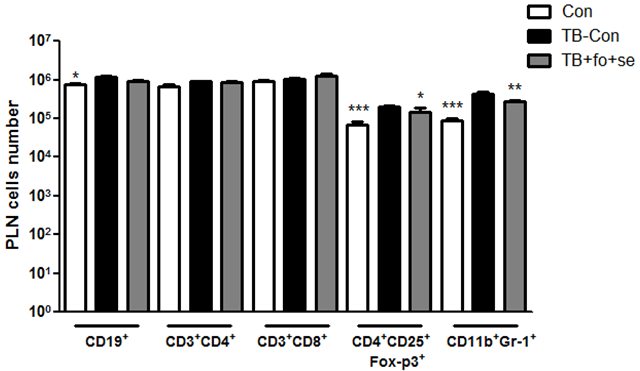

Supplement: Figure S2 — Effects of the combined nutrients on tumor induced immune suppressions in peripheral lymph nodes (experiment B). The combined nutrients were fed to mice for 36 days after tumor inoculation. PLN were homogenized and analyzed for CD19+ B, CD3+CD4+ T, CD3+CD8+ T cells, CD4+CD25+Fox-p3+ Tregs and CD11b+Gr-1+ MDSC in normal and tumor-bearing mice. Data show mean ± SD. of n = 7–8 mice/group and are representative of results from two independent experiments. *p<0.05, **p<0.01, and ***p<0.001 represent levels of significant differences among TB-Con mice. (TIF) [file pone.0052912.s002.tif]

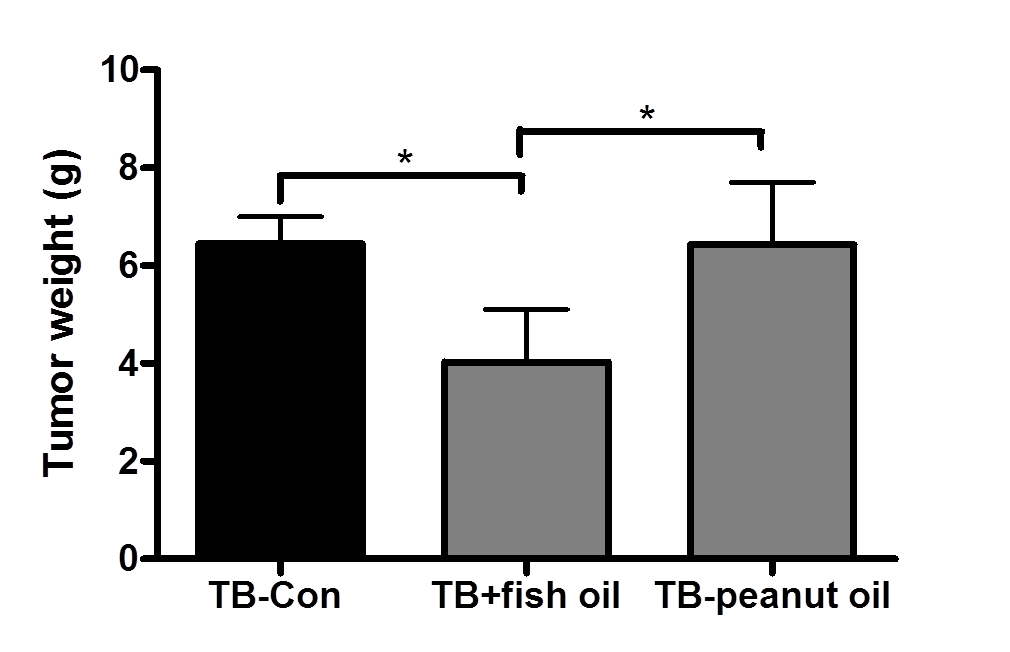

Supplement: Figure S3 — Effects of fish oil and peanut oil on tumor growth. Mice bearing line-1 tumors were treated p.o. with 150 mg/day of fish oil or peanut oil, and sacrificed by CO2 inhalation method on day 42. Data show mean ± SD. of n = 10–12 mice/group and are representative of results from three independent experiments. (TIF) [file pone.0052912.s003.tif]

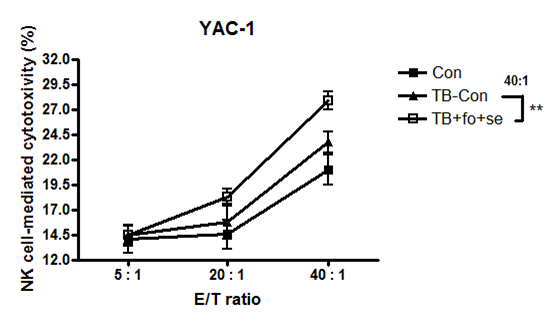

Supplement: Figure S4 — Effect of the combined nutrients on NK cytotoxicity in the spleen of BALB/c mice. Tumors in mice (n = 6–7 mice/group) were intervened with or without addition of fish oil and selenium yeast. After sacrifice, splenocytes from BALB/cByJ mice were used as the effector cells to test the protective effect of experimental diets for the NK-associated cytotoxicity against target cells (YAC-1). All values are means ± SD. **p<0.01 denote levels of significant differences between groups. (TIF) [file pone.0052912.s004.tif]
